# Case Report: Autoimmune Pulmonary Alveolar Proteinosis after COVID-19: A Report of Two Cases

**DOI:** 10.4269/ajtmh.22-0545

**Published:** 2023-04-24

**Authors:** Aatish Saraswat, Indramani Pandey, Simple Gupta, Manish Singh, Deepu Peter

**Affiliations:** ^1^ Department of Pathology, Armed Forces Medical College, Pune, India;; ^2^ Department of Pulmonary Medicine, Critical Care and Sleep Medicine, Armed Forces Medical College, Pune, India;; ^3^ Department of Ophthalmology, Armed Forces Medical College, Pune, India

## Abstract

Autoimmunity has been extensively established as a characteristic feature of the post-COVID-19 syndrome. There is evolving evidence of immune system dysregulation leading to the development of autoimmune phenomena in patients with COVID-19. This immune dysregulation may range from the production of autoantibodies to the new onset of rheumatic autoimmune diseases. An extensive literature search in databases from December 2019 to date revealed that no cases of autoimmune pulmonary alveolar proteinosis (PAP) were reported in post-COVID patients. In this context, we report a novel case series of two cases of new-onset autoimmune PAP in post-COVID patients, an entity that has not been described before. We recommend further studies to better understand this association between new-onset autoimmune PAP and SARS-CoV-2.

## INTRODUCTION

The COVID-19 pandemic caused by SARS-CoV-2 has led to an unprecedented burden on existing health facilities during and after the pandemic.^1^^,^^2^ Post-COVID-19 syndrome (PCS) as an entity is unfolding rapidly, as new cases are being reported as part of this disease spectrum; one part of this is the stimulation of autoantibodies in recovered patients leading to new-onset autoimmune diseases.^3^^–^^6^ Pulmonary alveolar proteinosis (PAP) is a rare lung disorder marked by aberrations in surfactant homeostasis. Broadly, it can be divided into primary (autoimmune), secondary, and congenital, out of which primary (autoimmune) PAP forms the significant bulk of diseases. Irrespective of the disease etiology, patients show alveolar surfactant deposition, which results in impaired gas exchange and may eventually lead to respiratory failure. There is adequate literature regarding certain viral infections (e.g., cytomegalovirus) leading to secondary PAP. However, the association between autoimmune PAP and SARS-CoV-2 has never been reported, unlike the well-established association between various viral infections and several autoimmune diseases.^7^^–^^9^

## CASE 1

**Figure 1.**
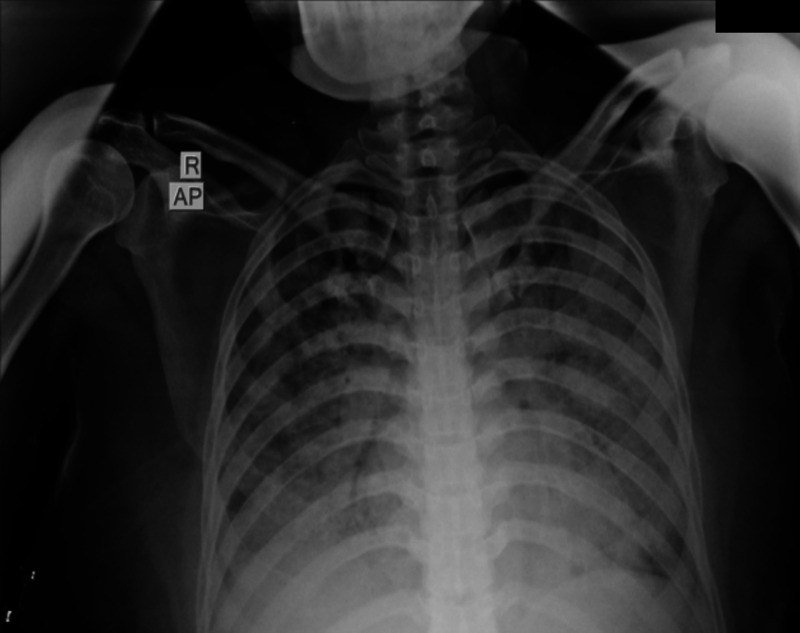
Chest radiograph (anterior–posterior view) of Case 1 showing bilaterally symmetrical diffuse alveolar opacities.

**Figure 2.**
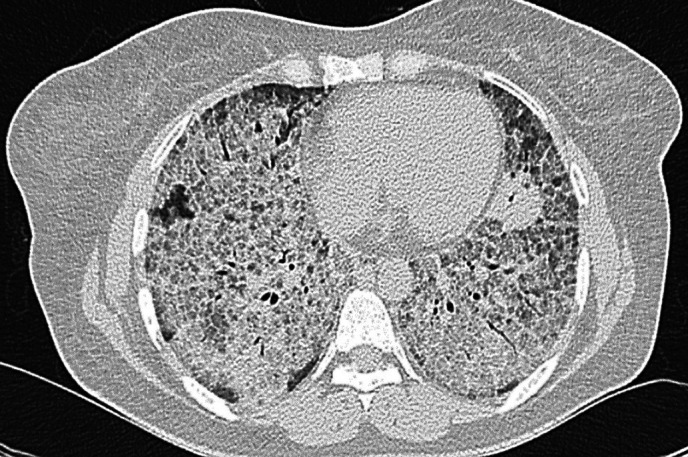
High-resolution computed tomography chest of Case 1, showing bilaterally diffuse ground-glass haziness with a network of smooth, interlobar septal thickening, with a characteristic “crazy-paving” appearance.

**Figure 3.**
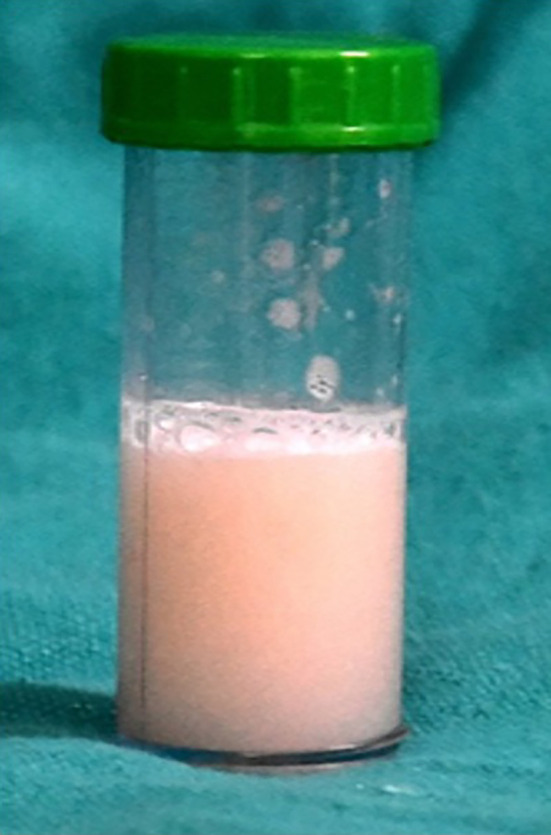
Milky white bronchoalveolar lavage, Case 1.

A 35-year-old female, unvaccinated for SARS CoV-2 and without prior comorbidities, presented to our hospital with a history of dry cough and progressive breathlessness (Modified Medical Research Council Dyspnea Scale [mMRC] grade 0–IV) over the past year. She had no history of exposure to toxins/drugs or features of any underlying connective tissue disease. On arrival, her nasal and throat swabs for SARS CoV-2 reverse transcriptase PCR were negative. Her vitals were as follows: pulse rate (PR) 122/minutes, respiratory rate (RR) 36 breaths/minutes, and oxygen saturation (SpO_2_) 76% at room air. On auscultation, she had fine crackles bilaterally, more at the bases. Other system examination was unremarkable. Chest radiograph showed bilateral diffuse alveolar opacities (Figure 1) and high-resolution computed tomography (CT) showed a bilateral crazy-paving appearance (Figure 2). Arterial blood gas (ABG) analysis revealed hypoxemia (partial pressure of oxygen [PaO_2_] 61 mm Hg and fraction of inspired oxygen [FiO_2_] of 0.40, PaO_2_/FiO_2_ [PF] ratio 153) with raised alveolar-arterial oxygen gradient (A-aDO_2_; 183.6 mm Hg). Because there was a high suspicion of SARS-CoV-2 infection, despite repeated negative RT PCR reports, SARS-CoV-2 antibody levels were tested, which were positive (IgG for SARS CoV-2), emphasizing past SARS CoV-2 infection. Her connective tissue disease profile was sent, among which antinuclear antibody by immunofluorescence (ANA by indirect fluorescence [IF]) was found to be positive (2+, midbody pattern) along with positive rheumatoid factor. Video bronchoscopy showed normal airways. However, bronchoalveolar lavage (BAL) was milky white (Figure 3) on gross appearance and microbiological tests were negative for bacteria, *Mycobacterium*, and fungus. Cytology with special staining of BAL displayed dense granular amorphous proteinaceous material forming small globules, which stained positive with periodic acid-Schiff (PAS) and PAS diastase (PAS-D) (Supplemental Figure 1). On the basis of the clinicoradiological findings and BAL analysis, she was diagnosed with PAP. To ascertain the etiology of PAP, secondary causes such as infections and malignancies were reasonably ruled out, and her blood samples were sent to Cincinnati Children’s Hospital (Cincinnati, OH), for anti–granulocyte-macrophage colony-stimulating factor (GM-CSF) antibodies, which were highly positive (55.6 µg/mL; normal range < 3.1 µg/mL); thus, she was diagnosed with autoimmune PAP. She was managed with two cycles of whole lung lavage under general anesthesia with single-lung ventilation (one lung at a time), after which her oxygenation improved. However, she had a rapid relapse of the disease within 4 weeks. Given refractory PAP, she was treated with anti CD20 monoclonal antibody (injected rituximab 1 g/dose, two doses given 2 weeks apart), followed by a repeat whole lung lavage after 4 weeks. Eventually, the patient responded well to the revised treatment and continues to be off oxygen support (PaO_2_ 66 mm Hg at FiO_2_ of 0.21, PF ratio 314) at follow-up.

## CASE 2

A 62-year-old female with no known comorbidities and a history of severe COVID-19 pneumonia 9 months earlier presented with complaints of dry cough and progressive breathlessness (mMRC grade I–III) for 4 months. On examination, she was in respiratory distress, and her vitals were as follows: PR 120/minutes, RR 32/minutes, and SpO_2_ 84% at room air. Auscultation revealed fine inspiratory crackles over both interscapular, infrascapular, and infraaxillary areas. Other system examinations were unremarkable. Her ABG analysis revealed hypoxemia (PaO_2_ 61 mm Hg at FiO_2_ of 0.30, PF ratio 203) with raised A-aDO_2_ (112.9 mm Hg). The chest radiograph showed bilateral alveolar opacities that was greater on the left side (Supplemental Figure 2). High-resolution CT revealed ground-glass opacities with a characteristic “crazy paving pattern” that was more conspicuous on the left side (Supplemental Figure 3). Further, during her ILD workup, serological studies revealed the presence of antinuclear antibody by immunofluorescence (ANA by IF 2+, coarse speckled pattern); SmD1, U1-RNP, and Ku autoantibodies; and raised aldolase with negative creatinine phosphokinase and myositis panel. She also underwent video bronchoscopy, BAL, and transbronchial lung biopsy. Bronchoalveolar lavage was milky white on gross appearance (Supplemental Figure 4) and displayed PAS and PAS-D positive amorphous proteinaceous material on cytology and special staining (Supplemental Figure 5). Her microbiological evaluation of BAL revealed no mycobacterial, fungal, or bacterial infection. Histopathological examination of the transbronchial lung biopsy (Supplemental Figure 6) showed normal lung parenchyma and granular eosinophilic amorphous PAS stain–positive material in many alveoli. She was diagnosed with PAP, and after secondary causes of PAP were ruled out, her blood samples for anti-GM-CSF antibodies were sent, which were positive (12.4 µg/mL, normal range < 3.1 µg/mL), suggesting autoimmune PAP. She was managed with whole lung lavage under general anesthesia with single-lung ventilation (one lung at a time), after which her oxygenation improved (PaO_2_ 66 mm Hg at FiO_2_ of 0.24, PF ratio 275).

## DISCUSSION

There is evidence that new-onset autoantibodies are formed during SARS-CoV-2 infection,^4^ which may persist after infection and increase with time in PCS.^10^ These autoantibodies may persist as latent autoimmunity long before they eventually manifest as overt autoimmune disease. According to one study that carried out serological screening for new-onset autoantibodies in patients with PCS, the prevalence of latent new-onset autoimmunity (at least one IgG autoantibody) was 83%, and the prevalence of new-onset poly-autoimmunity (two or more IgG autoantibodies) was 62%,^11^ even in the absence of severe clinical disease.^12^ However, the study also suggested that despite the high prevalence of autoimmunity among PCS patients, only a few developed overt autoimmune diseases.^11^ Various hypotheses have been presented to explain this new-onset autoimmunity. Molecular mimicry (human proteins with homologous regions to SARS-CoV-2, potentially function as autoantigens),^13^ bystander activation triggered by a hyperinflammatory state (often referred to as “cytokine storm” or “cytokine release syndrome”),^14^ viral persistence (polyclonal activation due to the constant presence of viral antigens driving immune-mediated injury) and formation of neutrophil extracellular traps^9^^,^^15^^,^^16^ are among the accepted hypothesis. In another study of post-COVID patients,^6^ vasculitis and arthritis were the most common autoimmune diseases, followed by idiopathic inflammatory myositis (IIM), systemic lupus erythematosus (SLE), and other autoimmune diseases. However, no case of autoimmune PAP has ever been reported in post-COVID patients. This study also revealed the relationship between autoimmune manifestation and severity of SARS-CoV-2 infection. Patients with mild COVID-19 mainly developed articular diseases (rheumatoid arthritis [RA], spondylarthritis [SpA], reactive arthritis [ReA]), whereas those with severe COVID-19 developed IIM, SLE, and vasculitis. In two cases of autoimmune PAP that we report here, we could not appreciate any such association with severity. One of the patients (Case 1) did not have any history of COVID-19 disease and was diagnosed with having a past SARS-CoV-2 infection based on raised antibody levels against SARS Cov-2. The other case (Case 2) had severe COVID-19. In addition, IIM and SLE presented predominantly during the acute phase of SARS-CoV-2 infection, whereas others presented later, suggesting different pathogenic mechanisms. In our cases, we could not ascertain the acute state of disease in Case 1, whereas Case 2 had a delayed manifestation. The same study revealed that the cases of IIM and SLE were more frequently reported in women, whereas seronegative SpA, ReA, RA, and vasculitis occurred more frequently in men. In our two cases of autoimmune PAP in post-COVID-19 patients, we noticed a female predominance that contrasts with what is seen in non–COVID-19-related autoimmune PAP.^17^ The management of the new-onset autoimmune diseases is essentially similar to that of the non–COVID-19-related counterparts, the exception being adult-onset Kawasaki disease, which proved to be more refractory to the usual treatment. In our cases, we had a discordant finding in which Case 1 behaved as refractory PAP and required the second line of treatment in the form of rituximab. In contrast, the response to the management in Case 2 was comparable to non–COVID-19 autoimmune PAP. Due to the limited information available regarding follow-up of post-COVID autoimmune diseases, it is still uncertain whether autoimmune diseases after COVID-19 have a similar long-term prognosis. In our experience at a tertiary care respiratory center, autoimmune PAP has been a rare entity over the past 2 decades. However, post COVID-19 pandemic, the emergence of such cases in succession cannot be considered chance alone and instead may hint toward a possible underlying association between the two diseases. The aim of this novel case series of two cases of post-COVID new-onset autoimmune PAP is to sensitize the treating physicians toward this new entity and enable them to make an early diagnosis of post-COVID new-onset autoimmune PAP. This would lead to prompt initiation of therapy, in turn resulting in successful recovery and preventing end-organ damage and fatality.

## Supplemental Materials


Supplemental materials

## References

[1] Zhu N , 2020. A novel coronavirus from patients with pneumonia in China, 2019. N Engl J Med 382: 727–733. 31978945 10.1056/NEJMoa2001017 PMC7092803

[2] World Health Organization *Coronavirus Disease (COVID-19)*. https://www.who.int/emergencies/diseases/novel-coronavirus-2019. Accessed March 12, 2023.

[3] Gazzaruso C Carlo Stella N Mariani G Nai C Coppola A Naldani D Gallotti P, 2020. High prevalence of antinuclear antibodies and lupus anticoagulant in patients hospitalized for SARS-CoV2 pneumonia. Clin Rheumatol 39: 2095–2097. 32462425 10.1007/s10067-020-05180-7 PMC7251560

[4] Chang SE , 2021. New-onset IgG autoantibodies in hospitalized patients with COVID-19. Nat Commun 12: 5417. 34521836 10.1038/s41467-021-25509-3 PMC8440763

[5] Fujii H , 2020. High levels of anti-SSA/Ro antibodies in COVID-19 patients with severe respiratory failure: a case-based review. Clin Rheumatol 39: 3171–3175. 32844364 10.1007/s10067-020-05359-y PMC7447083

[6] Gracia-Ramos AE Martin-Nares E Hernández-Molina G, 2021. New onset of autoimmune diseases following COVID-19 diagnosis. Cells 10: 3592. 34944099 10.3390/cells10123592 PMC8700122

[7] Smatti MK Cyprian FS Nasrallah GK Al Thani AA Almishal RO Yassine HM, 2019. Viruses and autoimmunity: a review on the potential interaction and molecular mechanisms. Viruses 11: E762. 10.3390/v11080762 PMC6723519 31430946

[8] Hussein HM Rahal EA, 2019. The role of viral infections in the development of autoimmune diseases. Crit Rev Microbiol 45: 394–412. 31145640 10.1080/1040841X.2019.1614904

[9] Winchester N Calabrese C Calabrese LH, 2021. The intersection of COVID-19 and autoimmunity: what is our current understanding? Pathog Immun 6: 31–54. 33969248 10.20411/pai.v6i1.417 PMC8097827

[10] Acosta-Ampudia Y Monsalve DM Rojas M Rodríguez Y Zapata E Ramírez-Santana C Anaya J-M, 2022. Persistent autoimmune activation and proinflammatory state in post-coronavirus disease 2019 syndrome. J Infect Dis 225: 2155–2162. 35079804 10.1093/infdis/jiac017 PMC8903340

[11] Anaya J-M , 2021. Post-COVID syndrome. A case series and comprehensive review. Autoimmun Rev 20: 102947. 34509649 10.1016/j.autrev.2021.102947 PMC8428988

[12] Liu Y , 2021. Paradoxical sex-specific patterns of autoantibody response to SARS-CoV-2 infection. J Transl Med 19: 524. 34965855 10.1186/s12967-021-03184-8 PMC8716184

[13] Mohkhedkar M Venigalla SSK Janakiraman V, 2021. Untangling COVID-19 and autoimmunity: identification of plausible targets suggests multi organ involvement. Mol Immunol 137: 105–113. 34242919 10.1016/j.molimm.2021.06.021 PMC8241658

[14] Camacho-Domínguez L Rodríguez Y Polo F Restrepo Gutierrez JC Zapata E Rojas M Anaya J-M, 2022. COVID-19 vaccine and autoimmunity. A new case of autoimmune hepatitis and review of the literature. J Transl Autoimmun 5: 100140. 35013724 10.1016/j.jtauto.2022.100140 PMC8730708

[15] Shah S Danda D Kavadichanda C Das S Adarsh MB Negi VS, 2020. Autoimmune and rheumatic musculoskeletal diseases as a consequence of SARS-CoV-2 infection and its treatment. Rheumatol Int 40: 1539–1554. 32666137 10.1007/s00296-020-04639-9 PMC7360125

[16] Zacharias H Dubey S Koduri G D’Cruz D, 2021. Rheumatological complications of Covid 19. Autoimmun Rev 20: 102883. 34237419 10.1016/j.autrev.2021.102883 PMC8256657

[17] Kariman K Kylstra JA Spock A, 1984. Pulmonary alveolar proteinosis: prospective clinical experience in 23 patients for 15 years. Lung 162: 223–231. 6492867 10.1007/BF02715650

