# Supplementary material for: Case Report: Autoimmune Pulmonary Alveolar Proteinosis after COVID-19: A Report of Two Cases
Source: Am J Trop Med Hyg. 2023 Apr 24;109(3):520–2. doi: 10.4269/ajtmh.22-0545 (PMC10484255; doi:10.4269/ajtmh.22-0545)

BAL - STAINS

Supplemental fig – 1; S1: BAL cytology - neutrophil predominant picture in a background of proteinaceous material on – Periodic Acid Schiff (Left) and May Grűnwald Giemsa stain (Right); case-1

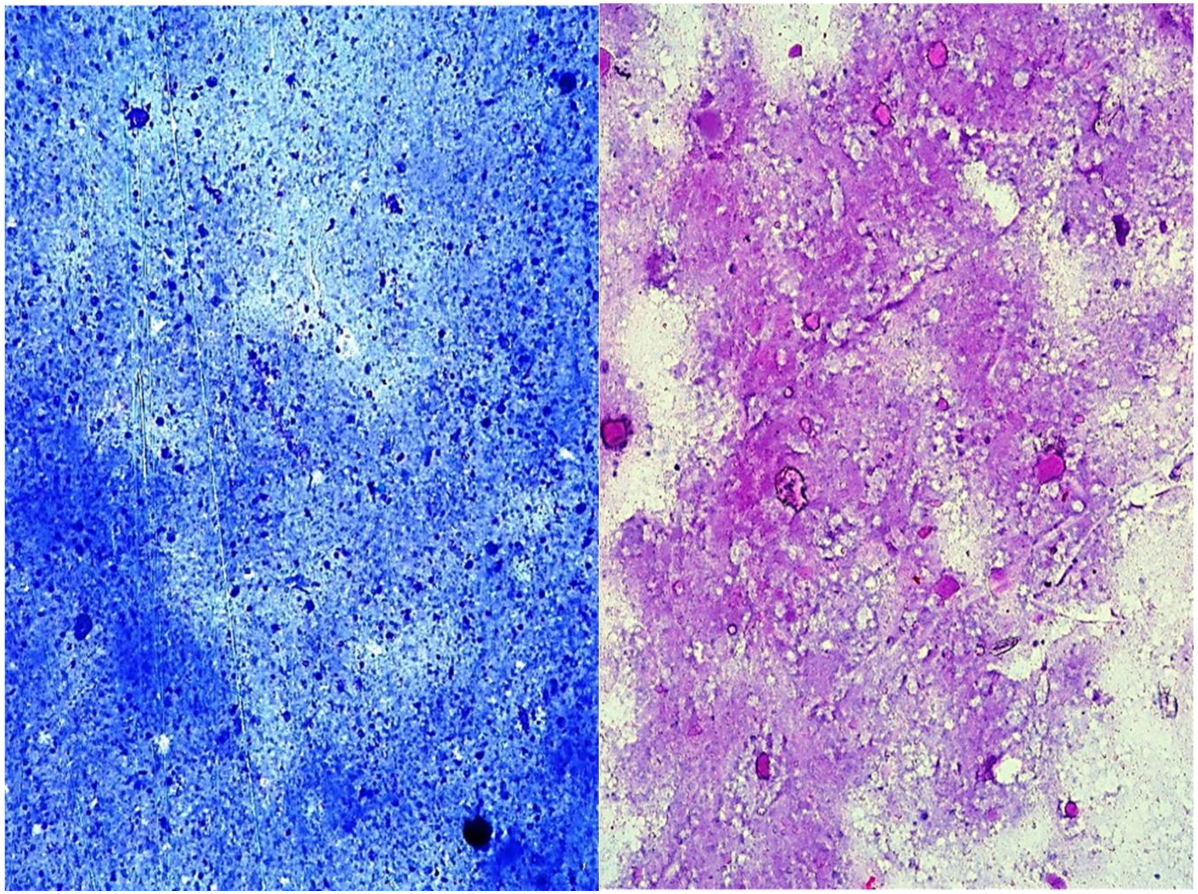

Chest Radiograph

Supplemental fig – 2; S2 : Chest radiograph (PA view) of case-2 showing bilateral central mid and lower zone predominant alveolar opacities more on the left side as compared to the right side

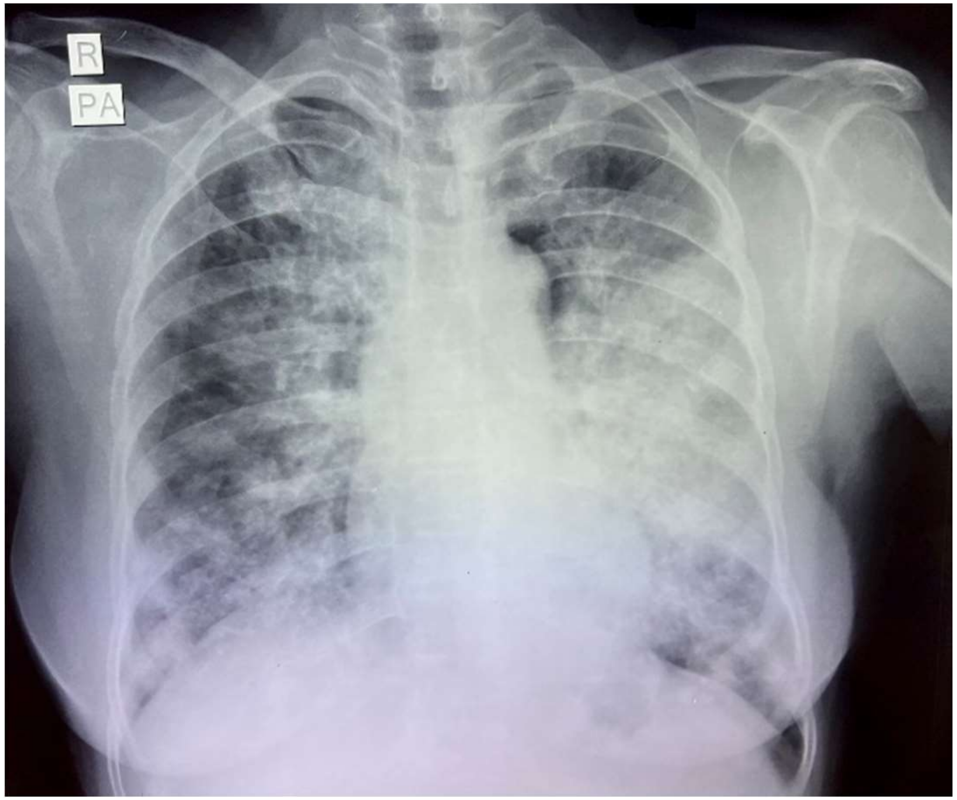

HRCT Chest

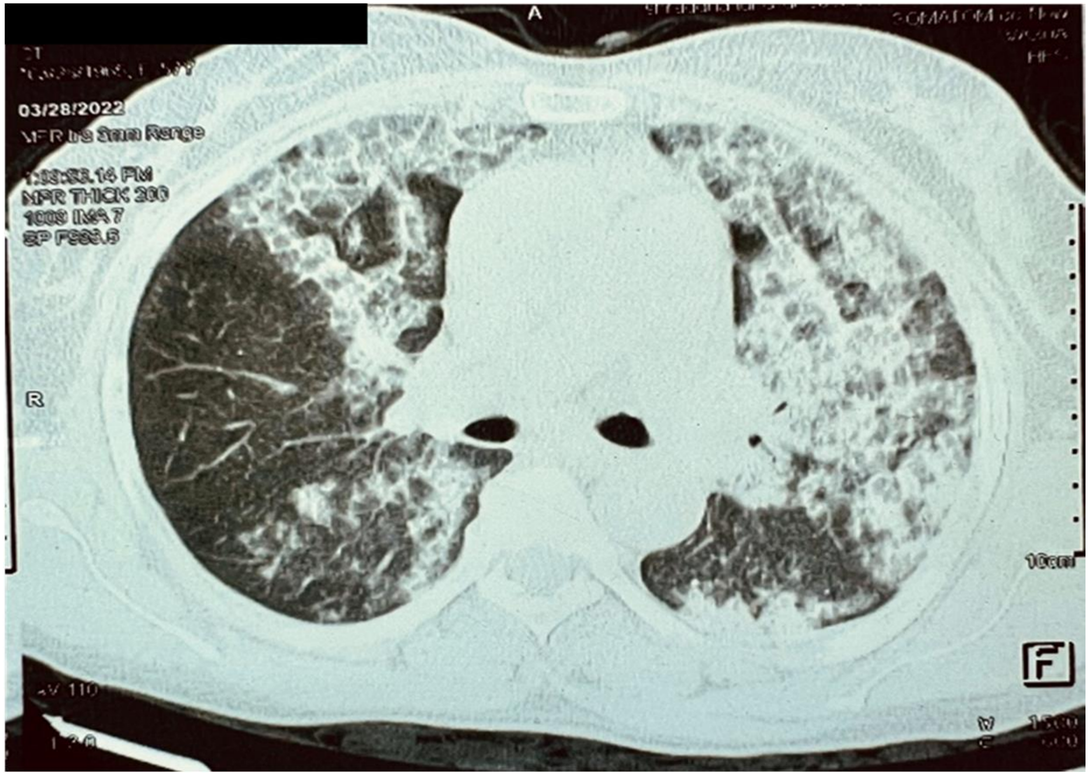

Supplemental fig – 3; S3 : HRCT Chest of case-2, showing bilateral diffuse ground-glass haziness with a network of smooth interlobar septal thickening with a crazy-paving appearance more on the left side as compared to the right side of the chest.

BAL

Supplemental fig – 4; S4 : Milky white bronchoalveolar lavage (BAL); case-2

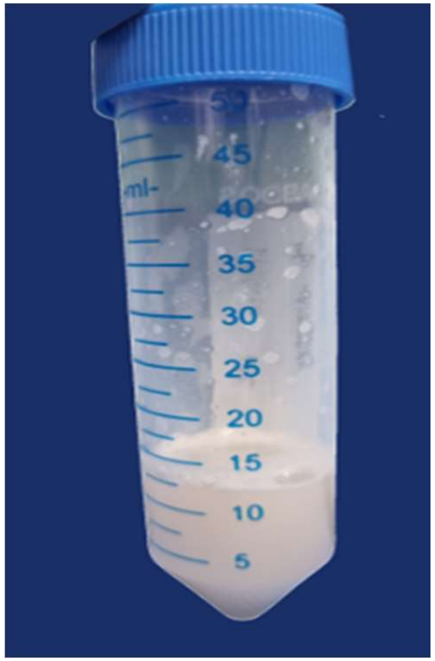

BAL - STAINS

Supplemental fig – 5; S5 : BAL cytology - neutrophil predominant picture in a background of proteinaceous material on – Periodic Acid Schiff (Left) and May Grűnwald Giemsa stain (Right); case-2

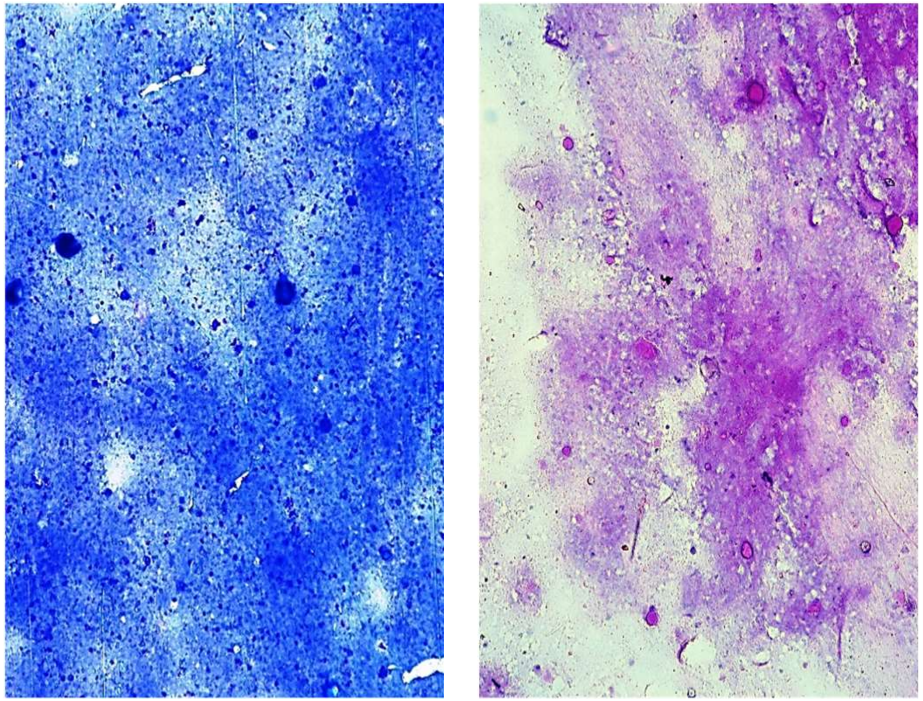

TBLB - stains

Supplemental fig – 6; S6 : Smears showing granular eosinophilic amorphous material in alveoli on - Periodic Acid Schiff stain – Magentophilic (Top left corner), H&E stain (Top right corner), Masson’s Trichrome stain (Bottom left corner), H&E stain (Bottom left corner).

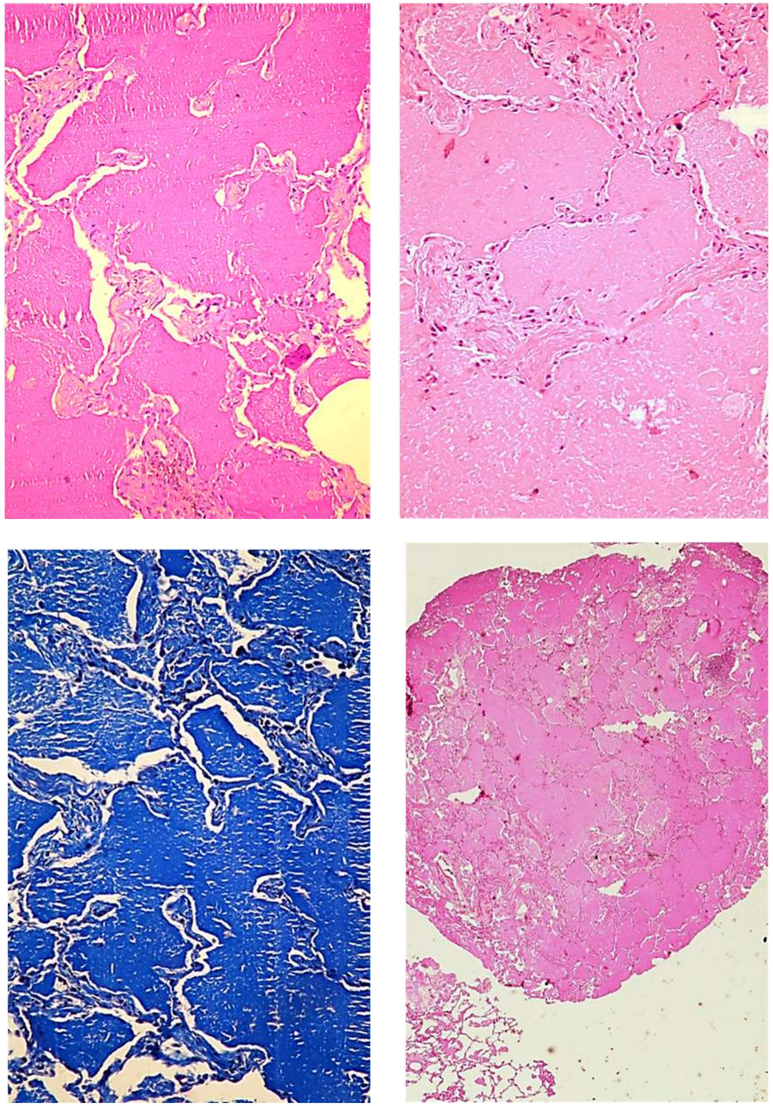

Supplement: Supplementary file 1 [file tpmd220545.SD1.pdf]
